# Spontaneous type 1 ECG and arrhythmic risk in Brugada syndrome: A meta-analysis of adjusted time-to-event data

**DOI:** 10.1016/j.hroo.2024.11.022

**Published:** 2024-11-28

**Authors:** Sotirios Chiotis, Luigi Pannone, Ioannis Doundoulakis, Domenico Giovanni Della Rocca, Stefanos Zafeiropoulos, Antonio Sorgente, Lorenzo Marcon, Giampaolo Vetta, Leonidas Koliastasis, Alvise Del Monte, Kazutaka Nakasone, Stavritsa T. Varvara, Mark La Meir, Ingrid Overeinder, Gezim Bala, Alexandre Almorad, Erwin Ströker, Juan Sieira, Dimitrios Tsiachris, Vasileios Vassilikos, Georgios Giannopoulos, Pedro Brugada, Andrea Sarkozy, Gian Battista Chierchia, Carlo de Asmundis

**Affiliations:** 1 Third University Department of Cardiology, Hippokration General Hospital, Aristotle University of Thessaloniki, Thessaloniki, Greece; 2 Heart Rhythm Management Centre, European Reference Networks GUARD-Heart, Universitair Ziekenhuis Brussel Heart Rhythm Research Brussels, Postgraduate Program in Cardiac Electrophysiology and Pacing, Vrije Universiteit Brussel, Brussels, Belgium; 3 Department of Cardiology, University Hospital of Zurich, Zurich, Switzerland; 4 First University Department of Cardiology, Hippokration General Hospital, National and Kapodistrian University of Athens, Athens, Greece; 5 Internal Medicine Department, 424 Military Hospital, Thessaloniki, Greece; 6 Cardiac Surgery Department, Universitair Ziekenhuis Brussel–Vrije Universiteit Brussel, Brussels, Belgium

**Keywords:** Brugada syndrome, Meta-analysis, Spontaneous type 1 ECG, Sudden cardiac death, Ventricular arrhythmias

## Abstract

**Background:**

Brugada syndrome (BrS) is associated with an increased risk of major arrhythmic events (MAEs), particularly in patients with a spontaneous type 1 electrocardiographic (ECG) pattern.

**Objective:**

Because previous meta-analyses used mainly crude or unadjusted data from observational studies, we conducted an updated meta-analysis on the prognostic role of spontaneous type 1 ECG in BrS patients combining adjusted and unadjusted data separately.

**Methods:**

We conducted a systematic search of PubMed and Cochrane Central Register of Controlled Trials from inception to May 2024. Studies providing hazard ratios for MAEs associated with spontaneous type 1 ECG in BrS patients were included.

**Results:**

Eighteen studies comprising 7238 patients were included, with 10 providing adjusted and 17 providing unadjusted data. Separate pooled analyses using a random-effects model demonstrated a significantly increased risk of MAEs in BrS patients with spontaneous type 1 ECG compared with those without, with a pooled adjusted hazard ratio (aHR) of 2.05 (95% CI 1.38–3.03) and an unadjusted hazard ratio of 2.97 (95% CI 2.04–4.34). Subgroup analysis revealed higher risks in studies with non-Asian populations and those including patients with no history of aborted cardiac arrest (aHR 2.36, 95% CI 1.35–4.11; and aHR 3.56, 95% CI 2.35–5.41, respectively) and a persistent significant risk in studies accounting for syncope as a covariate (aHR 2.01, 95% CI 1.24–3.27).

**Conclusion:**

Our analysis indicates that patients with BrS and spontaneous type 1 ECG are at higher risk of MAEs. This is consistent across various subgroups, including asymptomatic individuals.

## Graphical abstract

**Figure:**
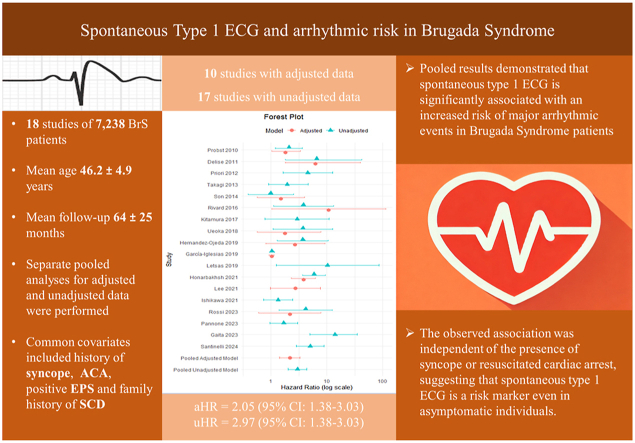

**Key Findings**

▪ Brugada syndrome patients with a spontaneous type 1 electrocardiography (ECG) experience a significantly elevated risk of major arrhythmic events (MAEs) compared with those without this ECG pattern, with an adjusted hazard ratio of 2.05 and an unadjusted hazard ratio of 2.97.
▪ The risk of MAEs associated with spontaneous type 1 ECG is notably pronounced in non-Asian populations and in patients with no history of aborted cardiac arrest. The increased risk persists across various subgroups, including studies that account for syncope as a confounding factor, with a similar elevated risk.
▪ Unadjusted effect estimates tend to overestimate the risk of MAEs, as they do not account for confounding factors. Adjusted data provide a more accurate measure of the actual risk, underlining the importance of multivariate analysis to accurately assess the prognostic value of spontaneous type 1 ECG.

## Introduction

Brugada syndrome (BrS) is an inherited cardiac arrhythmia syndrome characterized by a distinct electrocardiographic (ECG) pattern and an increased risk of sudden cardiac death (SCD).^1^ The hallmark of BrS is the presence of a type 1 ECG pattern, characterized by a coved-type ST-segment elevation in the right precordial leads (V1–V3), which can be spontaneous or induced by sodium-channel blockers.^2^

Risk stratification in BrS patients represents a major clinical challenge. While some individuals with BrS remain asymptomatic throughout their lives, others may experience syncope and ventricular arrhythmias (VAs), leading to SCD. The presence of a spontaneous type 1 ECG Brugada pattern has been identified as a key risk marker for major arrhythmic events (MAEs).^3^ To add complexity, spontaneous type 1 pattern may not be persistent, and its fluctuations can underestimate the risk in subjects with concealed spontaneous type 1 at presentation. A recent study showed that a spontaneous type 1 pattern can be detected in up to 12% of subjects with concealed BrS at presentation with ECG Holter monitoring. Its presence is associated with a worse arrhythmic prognosis, regardless of the time at detection.^4^

Because large registries with BrS patients have been published, several meta-analyses have been performed to evaluate the prognostic role of spontaneous type 1 ECG in the occurrence of MAEs.^5^^,^^6^ However, most of past literature used crude data from individual studies or provided unadjusted and a combination of adjusted and unadjusted effect estimates. As new evidence emerges, we sought to provide an updated and comprehensive assessment of the risk of MAEs in BrS patients with a spontaneous type 1 ECG, combining separately adjusted and unadjusted results from the published studies to date.

## Methods

The study was designed according to the recommendations of the PRISMA (Preferred Reporting Items for Systematic Reviews and Meta-Analyses) statement (Supplemental Table 1).^7^ All research was conducted according to a protocol registered in the PROSPERO database (registration number: CRD42024506439).

### Data sources and searches

PubMed and Cochrane Central Register of Controlled Trials were systematically searched from inception to May 2024 to identify all the relevant studies. A basic search strategy was developed for PubMed database and was accordingly modified for the other research databases. No restrictions of language, publication date, or age of the participants were applied. The search terms used were “Brugada,” “ECG,” and “spontaneous” or “type 1.” The search was conducted by 2 independent investigators (S.C. and I.D.) and the detailed search strategy is presented in Supplemental Table 2.

### Eligibility criteria

We included cohort studies including patients diagnosed with BrS. Studies were considered eligible if they met the following criteria: (1) BrS was defined as the presence of a spontaneous type 1 ECG or induced by pharmacological provocation with sodium-channel blockers, in accordance with the guidelines applicable at the time of each study^8^^,^^9^; (2) had a follow-up period of >6 months; and (3) presented hazard ratios (HRs) for spontaneous type 1 ECG, associated with MAEs during follow-up. MAEs were defined as sustained VAs, aborted cardiac arrest (ACA), appropriate implantable cardioverter-defibrillator (ICD) shocks, or SCD. The exclusion criteria were the following: (1) case reports/case series with <10 individuals, (2) certain publication types (letters, reviews, editorials, abstracts), and (3) duplicate data from the same registries. In case of duplicate publications, we included the study with the largest sample size or longest follow-up.

### Study selection and quality assessment

Search results were imported into a reference management software (EndNote X9; Clarivate). After removing duplicates, titles and abstracts were screened by 2 reviewers (S.C. and I.D.), with disagreements resolved by a third reviewer (L.P.). Full texts were assessed for quality using the Newcastle-Ottawa Scale, with a score of ≥7 indicating low bias.

### Data extraction

Data extraction was performed by 1 reviewer (S.C.) and verified by another (I.D.). Extracted items included study details (title, first author, year, location, design), participant characteristics (age, sex, ECG type, symptoms, history of VAs, family history of SCD), follow-up duration, electrophysiological characteristics, and MAEs during follow-up. Adjusted and unadjusted effect estimates along with their and confidence intervals (CIs) and confounding factors from the multivariate models were also extracted.

### Statistical analysis

Continuous variables were reported as mean ± SD or median (interquartile range). Summary estimates of continuous variables were reported as mean differences and categorical variables as HRs with 95% CIs. Two separate analyses were performed for the adjusted and unadjusted effect estimates.^10^ A DerSimonian-Laird random-effects meta-analysis was used due to anticipated heterogeneity between the studies. Results were visualized in a forest plot. Heterogeneity was assessed using Cochran’s Q test and quantified by I^2^ statistics. Subgroup, meta-regression, and leave-one-out analyses were conducted to examine sources of heterogeneity. Publication bias was assessed with a funnel plot and Egger’s regression test. Statistical analyses were performed using R version 4.1.1 (R Foundation for Statistical Computing) with meta and metafor packages.

## Results

### Search results

**Figure 1:**
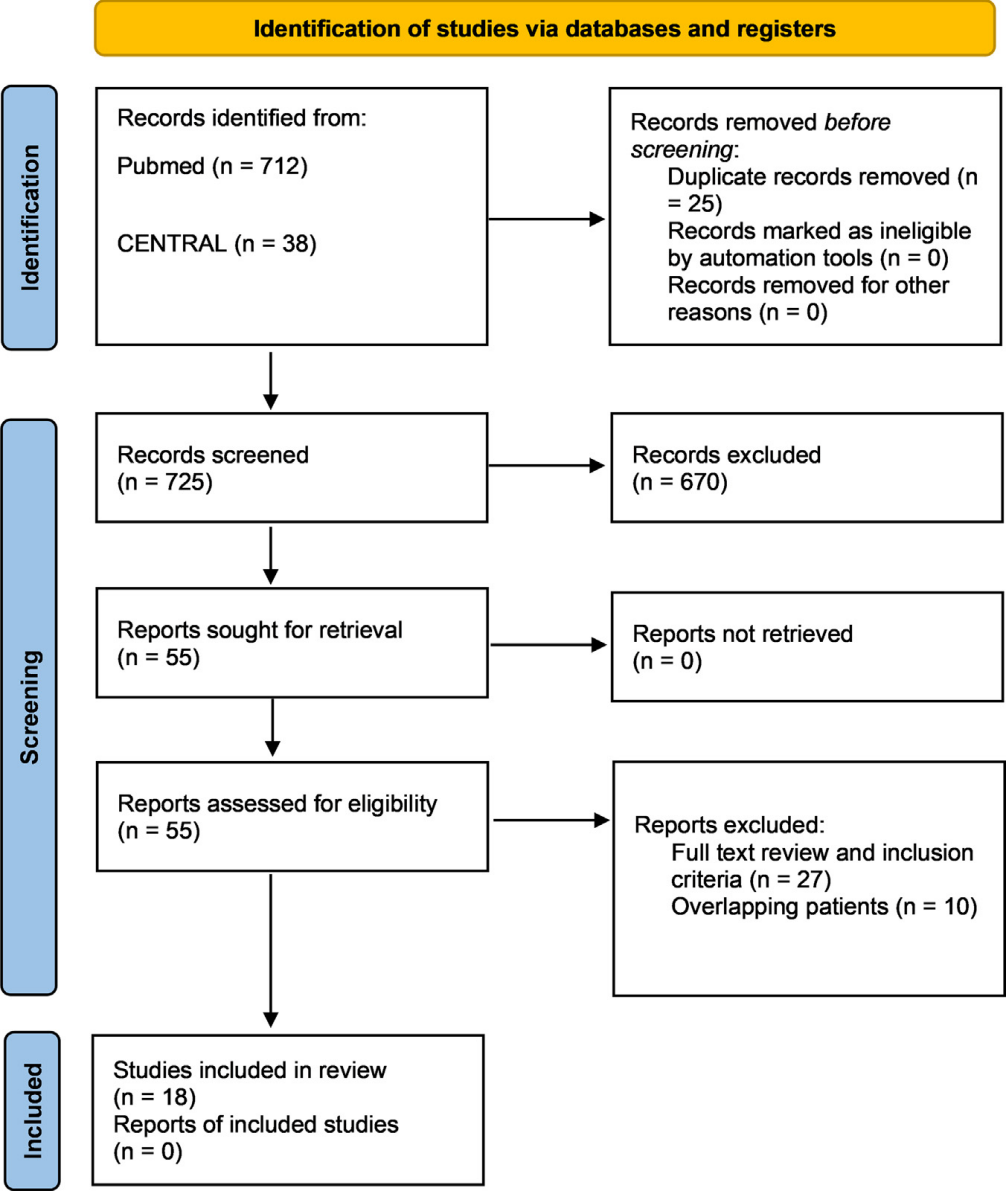
PRISMA (Preferred Reporting Items for Systematic Reviews and Meta-Analyses) flowchart of the study selection process.

After duplicate removal, 725 studies were evaluated based on their title and abstract. Out of the initial pool, 670 were excluded during the title/abstract screening phase due to irrelevant publication types (case reports, systematic reviews, editorials). Of the 55 cohort studies that were assessed for eligibility by full-text screening, 27 studies were excluded for not reporting the outcome of interest. Ten studies were excluded due to overlapping populations from the same registries. Two studies originating from Italy and 2 Japanese studies shared a common center, raising the possibility of an overlapping population. However, due to the multicenter design of these studies—the Italian studies involved 10 and 5 centers, respectively, and 1 Japanese study included 13 centers—the likelihood of significant overlap was minimal, so we decided to include both sets of studies to ensure a comprehensive analysis. Eventually, 18 studies with 7238 BrS patients were included in the systematic review and meta-analysis.^11^, ^12^, ^13^, ^14^, ^15^, ^16^, ^17^, ^18^, ^19^, ^20^, ^21^, ^22^, ^23^, ^24^, ^25^, ^26^, ^27^, ^28^ The study selection process is presented in Figure 1.

### Study and patient characteristics

Among the 18 studies, 6 were conducted at single-center locations, while the remaining studies were multicenter. Six studies consisted of Asian population. Six studies included patients with no history of ACA, while 1 study encompassed only asymptomatic patients. All studies presented data regarding the association of Spontaneous type 1 ECG and MAEs during follow-up, with periods ranging from 39 to 58 months.

The studies included a total population of 7653 patients with BrS. Overall, the majority were male (77%) with a mean age of 46.2 ± 4.9 years. In total, 35% of the patients were symptomatic at the initial presentation. A history of syncope and ACA was reported in 21% and 8% of the cases, respectively. Spontaneous type 1 ECG BrS pattern was present in 47% of the patients. Additionally, 21% reported a family history of sudden death. Detailed patient and study characteristics can be found in Table 1.

**Table 1:** Baseline characteristics of the included studies

Author, year	Origin	Subjects	n	Design	Age (y)	Male	Follow-up (mo)	Spontaneous type 1 ECG	Symptomatic	History of syncope	History of ACA/documented VA	Family history of SCD	ICD
Probst, 2010^11^	Multicenter (11 European hospitals in 4 countries/FINGER registry)	BrS patients with spontaneous or drug-induced type 1 ECG	1029	Retrospective	45 (35–55)	745 (72)	31.9 (14–54)	468 (45)	375 (36)	313 (30)	62 (6)	264 (26)	433 (42)
Delise, 2011^12^	Multicenter (5 Italian hospitals)	BrS patients with no history of cardiac arrest	320	Prospective	43 (33–54)	258 (81)	40 (20–67)	174 (54)	105 (33)	105 (33)	0 (0)	94 (29)	110 (34)
Priori, 2012^13^	Multicenter (12 Italian centers/PRELUDE registry)	BrS patients with no history of cardiac arrest	308	Prospective	47 ± 12	247 (80)	36 ± 8	171 (56)	65 (21)	65 (21)	0 (0)	NR	137 (44)
Takagi, 2013^14^	Single center (Osaka, Japan)	BrS probands with spontaneous or drug-induced type 1 ECG	460	Retrospective	51.9 ± 14	432 (94)	50 ± 32	290 (63)	193 (42)	109 (24)	84 (18)	107 (23)	NR
Son, 2014^15^	Multicenter (4 Korean hospitals)	BrS patients with ICD implantation	69	Retrospective	46.2 ± 13.5	68 (99)	59 ± 16.4	44 (64)	55 (80)	17 (25)	38 (55)	13 (19)	69 (100)
Rivard, 2016^16^	Multicenter (3 university centers in Quebec, Canada)	BrS patients with spontaneous or drug-induced type 1 ECG	105	Retrospective	46.2 ± 13.3	83 (79)	59.6 ± 16.4	87 (83)	47 (45)	37 (35)	10 (10)	28 (27)	56 (53)
Kitamura, 2017^17^	Single center (Tokyo, Japan)	BrS patients with spontaneous or drug-induced type 1 ECG	181	Prospective	51.9 ± 9.2	169 (93)	91.2 ± 69.6	86 (48)	66 (36)	42 (23)	24 (13)	41 (23)	73 (40)
Ueoka, 2018^18^	Single center (Okayama, Japan)	BrS patients undergoing provocative testing	245	Prospective	46.2 ± 13	240 (98)	113 ± 57	181 (74)	91 (37)	79 (32)	12 (5)	79 (32)	NR
Hernandez-Ojeda, 2020^21^	Single center (Barcelona, Spain)	BrS patients with history of syncope	135	Prospective	43.9 ± 13.9	96 (71)	92.4 ± 67.2	45 (33)	135 (100)	135 (100)	0 (0)	35 (26)	64 (47)
García-Iglesias, 2019^19^	Single center (Oviedo, Spain)	BrS patients with spontaneous or drug-induced type 1 ECG	337	Retrospective	40.9 ± 13.9	237 (70)	55.8 ± 39.35	43 (13)	110 (33)	95 (28)	15 (4)	159 (47)	47 (14)
Letsas, 2019^20^	Multicenter (9 hospitals in Greece)	BrS patients with no history of cardiac arrest	111	Prospective	45.4 ± 13.3	86 (77)	55.2 ± 42	49 (44)	37 (33)	37 (33)	0 (0)	7 (6)	34 (31)
Honarbakhsh, 2021^22^	Multicenter (16 centers from 8 countries)	BrS patients with no history of cardiac arrest	1110	Retrospective	51.8 ± 13.6	790 (71)	63.6 ± 48	388 (35)	352 (32)	352 (32)	0 (0)	235 (21)	172 (15)
Lee, 2021^24^	Multicenter (Hong Kong SAR)	BrS patients with spontaneous or drug-induced type 1 ECG (adult and young cohorts)	550 (adult cohort = 505, young cohort = 45)	Retrospective	58 ± 23	510 (93)	83 ± 80	413 (75)	218 (40)	175 (32)	43 (8)	45 (8)	143 (26)
Ishikawa, 2021^23^	Multicenter (Japan)	BrS probands undergoing genetic testing	415	Retrospective	46 ± 14	403 (97)	72 (1–279)∗	299 (72)	187 (45)	99 (24)	88 (21)	NR	241 (58)
Rossi, 2023^26^	Multicenter (5 centers in Italy)	BrS patients undergoing EPS	372	Retrospective	44 ± 15	257 (69)	48 (36–60)	185 (50)	170 (46)	94 (25)	0 (0)	105 (28)	89 (24)
Pannone, 2024^28^	Single center (Brussels, Belgium)	BrS patients undergoing reclassification of SCN5A variants	500	Retrospective	38.9 ± 16.9	245 (49)	84 (45–47)	87 (17)	188 (38)	159 (32)	29 (6)	75 (15)	197 (39)
Gaita, 2023^25^	Multicenter (2 centers in Rome, Italy)	Asymptomatic BrS patients with spontaneous or drug-induced type 1 ECG	1149	Prospective	45 ± 14	853 (74)	84 (48–132)	539 (47)	0 (0)	0 (0)	0 (0)	124 (11)	163 (14)
Santinelli, 2024^27^	Single center (Milan, Italy)	High-risk BrS patients with ICD undergoing epicardial ablation	257	Prospective	40.2 ± 10	174 (68)	27 (22–29)	43 (17)	257 (100)	176 (68)	81 (32)	89 (35)	257 (100)
Summary	—	—	7653	—	46.2 ± 4.9	5893 (77)	64 ± 25	3592 (47)	2651 (35)	2089 (27)	486 (6)	1500 (21)	2285 (30)

Values are mean ± SD, n (%), or median (interquartile range).

ACA = aborted cardiac arrest; BrS = Brugada syndrome; EPS = electrophysiology study; FINGER = France, Italy, Netherlands, Germany; ICD = implantable cardioverter-defibrillator; NR = not reported; PRELUDE = PRogrammed ELectrical stimUlation preDictive valuE; SCD = sudden cardiac death; VA = ventricular arrhythmia.

∗ Median (range).

In the included studies, the documentation of spontaneous Type 1 ECG was predominantly performed using standard 12-lead ECG at a single time point, with 2 studies employing Holter monitoring to unmask spontaneous type 1 patterns. The definitions of MAEs typically included documented VAs, appropriate ICD interventions, and SCD, with 1 study also considering cardiogenic syncope as a primary endpoint. A total of 11 studies were prospectively designed in which patients were followed longitudinally for the occurrence of MAEs, and 7 were retrospective with events being collected from past records. A summary of the definitions, methods of ECG documentation, and the prospective or retrospective nature of data collection for each study is provided in Supplemental Table 3.

Of the 18 studies included, 9 reported both adjusted HRs (aHRs) and unadjusted HRs (uHRs), 8 provided only uHRs, and 1 provided only aHRs. Common covariates in the multivariate models included syncope/symptomatic status, family history of SCD, inducible VAs in electrophysiology study (EPS), history of VA/ACA, male sex, and various ECG parameters. Detailed effect estimates along with the covariates used in multivariate models of the individual studies are presented in Supplemental Table 4.

### MAEs during follow-up

**Figure 2:**
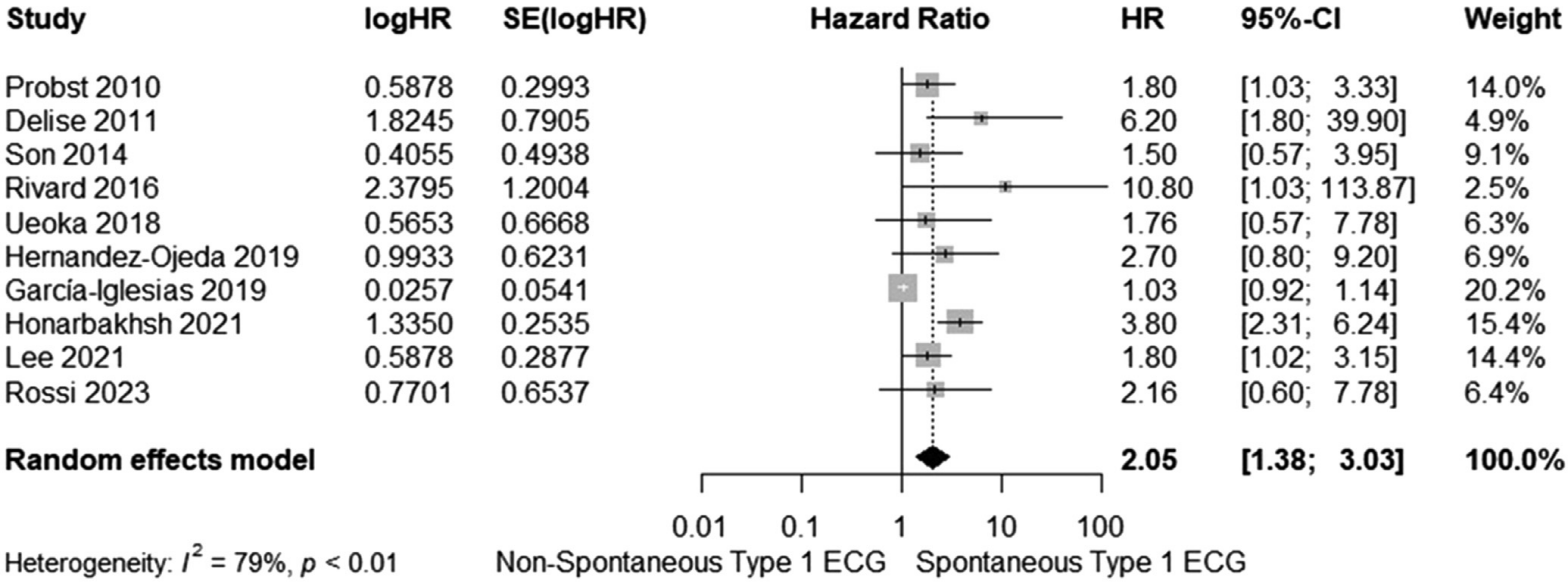
Forest plot of meta-analysis of the multivariate hazard ratios (HRs) for major arrhythmic events in Brugada syndrome patients with a spontaneous type 1 electrocardiography (ECG). CI = confidence interval.

**Figure 3:**
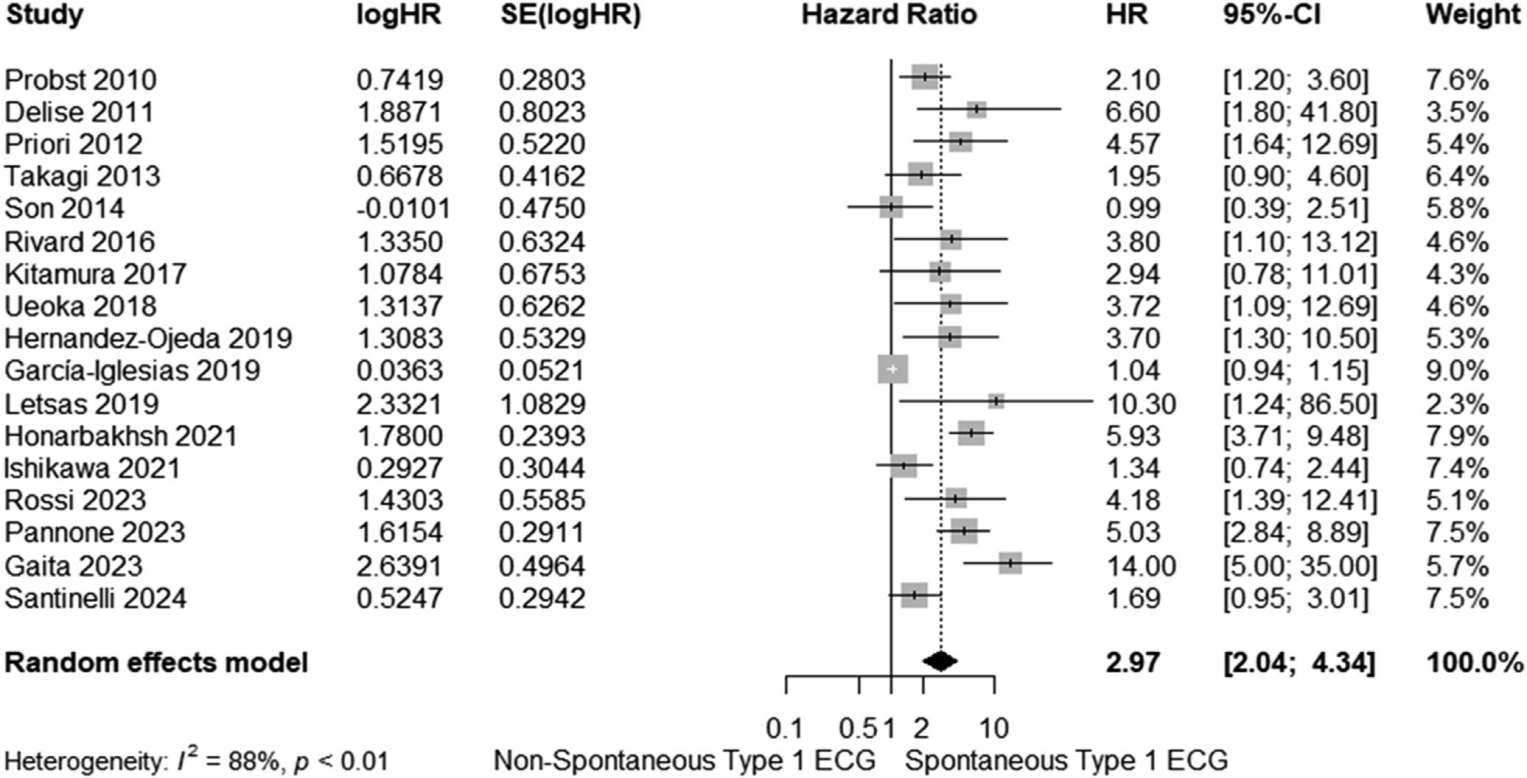
Forest plot of meta-analysis of the univariate hazard ratios (HRs) for arrhythmic events in Brugada syndrome patients with a spontaneous type 1 electrocardiography (ECG). CI = confidence interval.

Two separate analyses were performed for the multivariate and univariate models of each study, with 10 (4227 patients) and 17 studies (7103 patients) included in each analysis, respectively. As shown in Figures 2 and 3, the pooled analyses demonstrated a significantly increased risk of MAEs in BrS patients with spontaneous type 1 ECG Brugada pattern compared with those without, with a pooled aHR of 2.05 (95% CI 1.38–3.03, I^2^ = 79%) and uHR of 2.97 (95% CI 2.04–4.34, I^2^ = 88%), derived from the multivariate and univariate models, respectively.

### Subgroup analysis, sensitivity analysis, and meta-regression

Subgroup analysis, sensitivity analysis, and meta-regression were performed for the primary outcome of the adjusted models to identify potential sources of heterogeneity and to explore if differences in confounding factors between the studies affect the pooled effect estimate. Subgroup analysis based on the ethnicity (Asians vs non-Asians) and the inclusion of patients with a history of previous ACA (yes vs no) demonstrated significantly higher risk of MAEs in all subgroups with spontaneous type 1 ECG (Asians: aHR 1.72, 95% CI 1.09–2.72, I^2^ = 0%; non-Asians: aHR 2.36, 95% CI 1.35–4.11, I^2^ = 85%; history of ACA: aHR 1.47, 95% CI 1.02–2.12, I^2^ = 57%; no history of ACA: aHR 3.56, 95% CI 2.35–5.41, I^2^ = 0%) (Supplemental Figures 1 and 2). Notably, heterogeneity was decreased to 0% for the subgroups of Asians and the studies including populations with no history of ACA. Moreover, in the subgroup of studies including syncope as a confounding factor, results remained statistically significant (aHR 2.01, 95% CI 1.24–3.27, I^2^ = 83%) (Supplemental Figure 3).

A leave-one-out meta-analysis showed that omitting 1 study would reduce the I^2^ to 15% without heavily influencing the overall aHR (2.4, 95% CI 2.72–3.36) (Supplemental Figure 4). Meta-regression analysis demonstrated that proportions of male sex, spontaneous type 1 ECG, symptomatic status, history of syncope, history of ACA/VA, participant age, study design, methods for detection of spontaneous type 1 patterns, number of covariates, and the adjustment for syncope and ACA in the multivariate models of the individual studies were found to have no effect on the main analysis results (Supplemental Table 5). Factors including proportions of ICD implantation, family history of SCD, SCN5A mutation status, and positive EPS were reported in <10 studies and were not included in the meta-regression model.

### Publication bias

The publication bias of the studies included in the main meta-analysis was explored in funnel plots. Egger’s test showed significant publication bias (*P* = .005). To investigate the actual aHR in face of significant publication bias, a trim-and-fill analysis was performed, showing an aHR of 1.15 (95% CI 0.69–1.91), which might suggest an overestimation of the effect size obtained from the meta-analysis (Supplemental Figures 5 and 6).

### Risk of bias and grading of evidence

Using the Newcastle-Ottawa Scale, 7 studies scored more than 7 out of 9, indicating a low risk of bias. The remaining studies were considered to have a high risk of bias, primarily due to deficiencies in ensuring the outcome of interest was not present at the start of the study and in the comparability of cohorts. The quality of evidence was considered very low, due to inconsistency and high risk of publication bias (Supplemental Tables 6 and 7).

## Discussion

In this systematic review and meta-analysis, we demonstrated that BrS patients with spontaneous type 1 ECG have at least a 2-fold increased risk of MAEs when compared with those without this ECG pattern, regardless of other known risk factors. Notably, our findings establish that the predictive value of spontaneous type 1 ECG remains significant independently of the presence of syncope, as demonstrated by the subgroup analysis. Our results indicate that the pooled aHR was approximately 30% smaller than the pooled uHR (2.05 vs 2.97). Moreover, the pooled aHRs were found to be stronger in studies including non-Asian populations and in patients without history of ACA. This difference highlights that unadjusted effect estimates can overestimate true associations, as various factors may inflate the observed effect size. The majority of published systematic reviews and meta-analyses have relied on unadjusted estimates or crude event data, failing to account for potential confounding effects, thereby likely biasing the strength or direction of the associations.^5^^,^^6^^,^^29^ Our findings provide robust evidence supporting an independent relationship between spontaneous type 1 ECG and MAEs in BrS patients. These results emphasize the critical importance of considering potential confounding factors in such analyses to ensure accurate risk assessment.

Since BrS was first identified, extensive efforts have been made to identify high-risk patients for arrhythmic events and SCD. Syncope and history of ACA^6^^,^^30^ are the best-documented high-risk features. Other risk factors have been studied, without definitive evidence of an increased risk for MAEs. The prognostic value of familial SCD remains controversial, with studies presenting varying results.^6^^,^^31^ Similarly, the usefulness of EPS has been debated for a long time, mainly due to inconsistent protocols used across different studies, resulting in a class IIb recommendation for asymptomatic individuals.^9^ Recently, several ECG risk factors have been proposed, including fragmented QRS, early repolarization pattern, aVR sign, T-peak-to-T-end interval, and the presence of an S-wave in lead I.^32^ Moreover, loss-of-function SCN5A variants confer a greater risk of VA.^28^^,^^33^ Throughout the literature, spontaneous type 1 ECG has consistently been proposed as a major risk marker for arrhythmic events. This simple, noninvasive tool offers valuable insights into the electrophysiologic characteristics of BrS patients, highlighting intrinsic depolarization and repolarization abnormalities.^3^^,^^6^^,^^13^^,^^34^^,^^35^

Risk stratification in BrS remains challenging, especially for asymptomatic patients. The France, Italy, Netherlands, Germany registry results did not show a statistically significant prognostic role for spontaneous type 1 ECG in the asymptomatic individuals (HR 2.00, *P* = 0.26). In contrast, a study by Gaita and colleagues^25^ involving the largest cohort of asymptomatic BrS individuals (1149 participants) demonstrated that patients with spontaneous type 1 ECG had a 14-fold higher risk of arrhythmic events compared with those with drug-induced Brugada pattern, with a 3% vs 0.16% occurrence of arrhythmic events over a median follow-up of 6 years. This significant risk difference could be attributed to the comprehensive evaluation methods in the latter study, including multiple ECGs and repeated 24-hour Holter monitoring. These methods likely identified a subset of patients with spontaneous type 1 ECG that might have been initially classified as drug-induced, due to the high variability of the Brugada ECG pattern over time. Most of the studies included in this systematic review relied on single-time-point 12-lead ECGs to detect the spontaneous type 1 pattern, with only 2 using 24-hour Holter monitoring, one of which was included in the meta-analysis of adjusted effect estimates.^26^ Omitting this study did not significantly alter the pooled HR or heterogeneity (HR 2.05, 95% CI 1.35–3.12, I^2^ = 81%) (Supplemental Figure 4). However, Holter monitoring remains a valuable tool for increasing the detection of spontaneous type 1 ECG patterns, as it can capture intermittent patterns that may be missed by single-time-point ECGs. Incorporating Holter monitoring into routine clinical practice could enhance early detection and improve risk stratification in BrS, especially in asymptomatic individuals.^4^^,^^25^

A leave-one-out sensitivity analysis indicated that excluding the study by Rossi and colleagues,^26^ which used Holter monitoring, did not significantly alter the pooled HR, suggesting that the variability in ECG detection methods had minimal impact on the overall findings. Our meta-analysis, in line with this finding, highlights the independent role of spontaneous type 1 ECG in predicting MAEs. Most studies included in the multivariate analysis were adjusted for symptomatic status and/or history of syncope, emphasizing the necessity for a thorough evaluation of asymptomatic BrS patients to identify the presence of spontaneous type 1 ECG pattern.

This study features several strengths, representing one of the most comprehensive meta-analyses on the prognostic role of spontaneous type 1 ECG in BrS, incorporating the largest number of patients so far. By separately pooling aHRs and uHRs, it provides a robust estimate of risk, accounting for potential confounding factors.

### Limitations

Several limitations must be acknowledged. Our meta-analysis relied on the adjusted effect estimates available in studies that used different sets of covariates, which might have contributed to the variations observed. Moreover, due to the observational nature of the included studies, our analysis is subject to selection and recall bias. Furthermore, significant heterogeneity was observed among the included studies, which could affect the generalizability of the findings. Despite subgroup analyses, some degree of heterogeneity remained unexplained, potentially impacting the precision of the pooled risk estimates. Finally, the presence of significant publication bias, as indicated by Egger’s test, suggests that the pooled effect size may be overestimated. The trim-and-fill results highlight a critical limitation in our findings, as the nonsignificant aHR suggests that the true effect of spontaneous type 1 ECG may be weaker than initially observed. Therefore, while our findings indicate an elevated risk, the strength of the evidence is compromised, and future research, particularly studies that include unpublished or negative results, will be mandatory to confirm this association.

## Conclusion

This meta-analysis demonstrates the significant prognostic value of spontaneous type 1 ECG in BrS patients, highlighting its association with an increased risk of MAEs. Despite substantial heterogeneity among studies, spontaneous type 1 ECG consistently emerged as a critical marker for identifying high-risk individuals. This underscores the importance of thorough evaluations, particularly in asymptomatic patients, using serial 12-lead ECG Holter recordings at various intercostal spaces to detect spontaneous ECG patterns. Notably, the observed association was independent of the presence of syncope or resuscitated SCD, suggesting that spontaneous type 1 ECG is a significant risk marker even in asymptomatic individuals.
