# Supplementary material for: Spontaneous type 1 ECG and arrhythmic risk in Brugada syndrome: A meta-analysis of adjusted time-to-event data
Source: Heart Rhythm O2. 2024 Nov 28;6(2):195–203. doi: 10.1016/j.hroo.2024.11.022 (PMC11993785; doi:10.1016/j.hroo.2024.11.022)
Supplement: Supplementary Data [file mmc1.docx]

Supplementary material:

**Spontaneous Type 1 ECG and arrhythmic risk in Brugada Syndrome: a meta-analysis of adjusted time-to-event data**

**Contents**

[**Supplementary table 1:** Preferred Reporting Items for Systematic reviews and Meta-Analysis (PRISMA) checklist 3](#_Toc179733240)

[**Supplementary table 2**: Search terms 6](#_Toc179733241)

[**Supplementary table 3**. Overview of documentation of Spontaneous Type 1 ECG, definition of Major Arrhythmic Events and outcome data collection in the included studies. 8](#_Toc179733242)

[**Supplementary table 4**. Results of univariate and multivariate models of the individual studies 9](#_Toc179733244)

[**Supplementary figure 1:** Forest plot of subgroup analysis depending on the inclusion of patients with history of Aborted Cardiac Arrest in the included studies. 12](#_Toc179733245)

[**Supplementary figure 2:** Forest plot of subgroup analysis depending on the ethnicity of the BrS population in the included studies. 13](#_Toc179733246)

[**Supplementary figure 3:** Forest plot of subgroup analysis depending on the inclusion of syncope as a confounding factor in the included studies. 14](#_Toc179733247)

[**Supplementary figure 4:** Leave-one-out sensitivity analysis. 15](#_Toc179733248)

[**Supplementary table 5:** Meta-regression analysis* 16](#_Toc179733249)

[**Supplementary figure 5:** Funnel plot of the studies included in the meta-analysis of aHRs. 17](#_Toc179733250)

[**Supplementary figure 6.** Funnel plot of trim-and-fill method to correct publication bias. 18](#_Toc179733251)

[**Supplementary table 6:** Quality assessment using the Newcastle-Ottawa Scale (NOS) 19](#_Toc179733252)

[**Supplementary table 7:** Grading of evidence for the primary outcome 20](#_Toc179733253)

## **Supplementary table 1:** Preferred Reporting Items for Systematic reviews and Meta-Analysis (PRISMA) checklist

| **Section/topic** | **#** | **Checklist item** | **Reported on page #** |
| --- | --- | --- | --- |
| **TITLE** | | |  |
| Title | 1 | Identify the report as a systematic review, meta-analysis, or both. | 1 |
| **ABSTRACT** | | |  |
| Structured summary | 2 | Provide a structured summary including, as applicable: background; objectives; data sources; study eligibility criteria, participants, and interventions; study appraisal and synthesis methods; results; limitations; conclusions and implications of key findings; systematic review registration number. | 2 |
| **INTRODUCTION** | | |  |
| Rationale | 3 | Describe the rationale for the review in the context of what is already known. | 5 |
| Objectives | 4 | Provide an explicit statement of questions being addressed with reference to participants, interventions, comparisons, outcomes, and study design (PICOS). | 5 |
| **METHODS** | | |  |
| Protocol and registration | 5 | Indicate if a review protocol exists, if and where it can be accessed (e.g., Web address), and, if available, provide registration information including registration number. | 6 |
| Eligibility criteria | 6 | Specify study characteristics (e.g., PICOS, length of follow-up) and report characteristics (e.g., years considered, language, publication status) used as criteria for eligibility, giving rationale. | 6,7 |
| Information sources | 7 | Describe all information sources (e.g., databases with dates of coverage, contact with study authors to identify additional studies) in the search and date last searched. | 6 |
| Search | 8 | Present full electronic search strategy for at least one database, including any limits used, such that it could be repeated. | Supplementary table 2 |
| Study selection | 9 | State the process for selecting studies (i.e., screening, eligibility, included in systematic review, and, if applicable, included in the meta-analysis). | 7 |
| Data collection process | 10 | Describe method of data extraction from reports (e.g., piloted forms, independently, in duplicate) and any processes for obtaining and confirming data from investigators. | 7 |
| Data items | 11 | List and define all variables for which data were sought (e.g., PICOS, funding sources) and any assumptions and simplifications made. | 7,8 |
| Risk of bias in individual studies | 12 | Describe methods used for assessing risk of bias of individual studies (including specification of whether this was done at the study or outcome level), and how this information is to be used in any data synthesis. | 7 |
| Summary measures | 13 | State the principal summary measures (e.g., risk ratio, difference in means). | 8 |
| Synthesis of results | 14 | Describe the methods of handling data and combining results of studies, if done, including measures of consistency (e.g., I^2^) for each meta-analysis. | 8 |
| Risk of bias across studies | 15 | Specify any assessment of risk of bias that may affect the cumulative evidence (e.g., publication bias, selective reporting within studies). | 7 |
| Additional analyses | 16 | Describe methods of additional analyses (e.g., sensitivity or subgroup analyses, meta-regression), if done, indicating which were pre-specified. | 7,8 |
| **RESULTS** | | |  |
| Study selection | 17 | Give numbers of studies screened, assessed for eligibility, and included in the review, with reasons for exclusions at each stage, ideally with a flow diagram. | 9 |
| Study characteristics | 18 | For each study, present characteristics for which data were extracted (e.g., study size, PICOS, follow-up period) and provide the citations. | 9, Table 1 |
| Risk of bias within studies | 19 | Present data on risk of bias of each study and, if available, any outcome level assessment (see item 12). | 12 |
| Results of individual studies | 20 | For all outcomes considered (benefits or harms), present, for each study: (a) simple summary data for each intervention group (b) effect estimates and confidence intervals, ideally with a forest plot. | 10,11, Table 1 |
| Synthesis of results | 21 | Present results of each meta-analysis done, including confidence intervals and measures of consistency. | 10,11, Figure 1, Figure 2 |
| Risk of bias across studies | 22 | Present results of any assessment of risk of bias across studies (see Item 15). | Supplementary table 4 |
| Additional analysis | 23 | Give results of additional analyses, if done (e.g., sensitivity or subgroup analyses, meta-regression [see Item 16]). | 10, 11, Supplementary figure 1-6, supplementary table 3 |
| **DISCUSSION** | | |  |
| Summary of evidence | 24 | Summarize the main findings including the strength of evidence for each main outcome; consider their relevance to key groups (e.g., healthcare providers, users, and policy makers). | 12-15 |
| Limitations | 25 | Discuss limitations at study and outcome level (e.g., risk of bias), and at review-level (e.g., incomplete retrieval of identified research, reporting bias). | 14 |
| Conclusions | 26 | Provide a general interpretation of the results in the context of other evidence, and implications for future research. | 15 |
| **FUNDING** | | |  |
| Funding | 27 | Describe sources of funding for the systematic review and other support (e.g., supply of data); role of funders for the systematic review. | 15 |

## **Supplementary table 2**: Search terms

|  | | |
| --- | --- | --- |
| *Search entry Pubmed* | | |
| Search | Entry terms | Result |
| #1 | Brugada | 7,506 |
| #2 | ECG | 256,502 |
| #3 | Spontaneous | 488,116 |
| #4 | Type 1 | 1,536,220 |
| #5 | #1 AND #2 AND (#3 OR#4) | 712 |
| *Search entry Cochrane* | | |
| Search | Entry terms | Result |
| #1 | Brugada | 173 |
| #2 | ECG | 17,148 |
| #3 | Spontaneous | 24,118 |
| #4 | Type 1 | 1,414,913 |
| #5 | #1 AND #2 AND (#3 OR#4) | 38 |
| Summary | | |
| Combined search results from all search engines | | 750 |
| After exclusion of duplicates, review articles, case reports, conference publications, and editorials. | | 725 |

## **Supplementary table 3**. Overview of documentation of Spontaneous Type 1 ECG, definition of Major Arrhythmic Events and outcome data collection in the included studies.

| **Study** | **Methods for Detecting Spontaneous Type 1 ECG** | **Definition of MAEs** | **Prospective Data Collection** |
| --- | --- | --- | --- |
| **Probst 2010** | Single time-point 12-lead ECG | VAs, appropriate ICD interventions, SCD | Yes |
| **Delise 2011** | Single time-point 12-lead ECG | SCD, VAs recorded by ICD or conventional ECG | Yes |
| **Priori 2012** | Single time-point 12-lead ECG | VF, appropriate ICD interventions | Yes |
| **Takagi 2013** | Single time-point 12-lead ECG | SCD, VF | Yes |
| **Son 2014** | Single time-point 12-lead ECG | Appropriate ICD interventions | No |
| **Rivard 2016** | Single time-point 12-lead ECG | SCD, appropriate ICD interventions | No |
| **Kitamura 2017** | Single time-point 12-lead ECG | Documented VAs | No |
| **Ueoka 2018** | Single time-point 12-lead ECG | Documented VAs, SCD, appropriate ICD interventions | Yes |
| **Hernandez-Ojeda 2019** | Single time-point 12-lead ECG | Cardiogenic syncope, VAs, SCD | No |
| **Garcia-Iglesias 2019** | Single time-point 12-lead ECG | SCD, appropriate ICD interventions | No |
| **Letsas 2019** | Single time-point 12-lead ECG | SCD, appropriate ICD interventions | Yes |
| **Honarbakhsh 2021** | Single time-point 12-lead ECG | SCD, documented sustained VAs, appropriate ICD interventions | Yes |
| **Lee 2021** | Single time-point 12-lead ECG | Documented VAs, appropriate ICD interventions | No |
| **Ishikawa 2021** | Single time-point 12-lead ECG | SDC, CA, documented VAs, appropriate ICD interventions | No |
| **Rossi 2023** | Single time-point 12-lead ECG, 12-lead 24-h Holter monitoring | SCD, resuscitated CA, appropriate ICD interventions | Yes |
| **Pannone 2023** | Single time-point 12-lead ECG | Appropriate ICD interventions, SCD, documented VAs | Yes |
| **Gaita 2023** | Single time-point 12-lead ECG, 12-lead 24-h Holter monitoring | SCD, Appropriate ICD interventions | Yes |
| **Santinelli 2024** | Single time-point 12-lead ECG | Appropriate ICD interventions, VF storms | Yes |

## VA, Ventricular arrhythmia; ICD, Implantable Cardioverter-Defibrillator; SCD, Sudden Cardiac Death; VF, Ventricular Fibrillation; CA, Cardiac Arrest

## **Supplementary table 4**. Results of univariate and multivariate models of the individual studies

| **Author, Year** | **HR (Univariate Model)** | **95% CI** | **HR (Multivariate Model)** | **95% CI** | **Covariates** |
| --- | --- | --- | --- | --- | --- |
| **Probst 2010** | 2.1 | 1.2-3.6 | 1.8 | 1.03-3.33 | ACA, syncope, positive EPS, age, gender |
| **Delise 2011** | 6.6 | 1.8-41.8 | 6.2 | 1.8-39.9 | Syncope |
| **Priori 2012** | 4.57 | 1.64-12.69 | - | - | - |
| **Takagi 2013** | 1.95 | 0.9-4.6 | - | - | - |
| **Son 2014** | 0.99 | 0.39-2.51 | 1.5 | 0.57-3.95 | syncope, ACA, family history of SCD |
| **Rivard 2016** | 3.8 | 1.1-13.12 | 10.8 | 1.03-113.87 | Tp-e duration >100 ms, QRS duration inV6 >110 ms |
| **Kitamura 2017** | 2.94 | 0.78-11.01 | - | - | - |
| **Ueoka 2018** | 3.72 | 1.09-12.69 | 1.76 | 0.57-7.78 | Symptomatic status (syncope, VT/VF), PQ>235ms, QRS>132ms, ST>0.3mV, SCB-induced VAs |
| **Hernandez-Ojeda 2019** | 3.7 | 1.3-10.5 | 2.7 | 0.8-9.2 | Cardiogenic syncope |
| **García-Iglesias 2019** | 1.037 | 0.936-1.148 | 1.026 | 0.923-1.141 | Total ST power, gender male, Family history of SCD, syncope, positive EPS, SCN5A mutation |
| **Letsas 2019** | 10.3 | 1.24-86.5 | - | - | - |
| **Honarbakhsh 2021** | 5.93 | 3.71-9.48 | 3.8 | 2.31-6.24 | Arrhythmia-related syncope, diagnosis by family screening of SCD, ER pattern in peripheral leads, type 1 ECG in peripheral leads |
| **Lee2021** | - | - | 2.74 | 0.98-7.65 | Initially asymptomatic, initially VA, P wave axis, S wave amplitude in V1, R wave amplitude in V5 |
| **Ishikawa 2021** | 1.34 | 0.74-2.44 | - | - | - |
| **Rossi 2023** | 4.18 | 1.39-12.41 | 2.16 | 0.6-7.78 | Difference in the refractory period between RVOT and RV apex (ΔRPRVOT-apex) >60 ms. |
| **Pannone 2023** | 1.69 | 0.95-3.01 | - | - | - |
| **Gaita 2023** | 14 | 5-35 | - | - | - |
| **Santinelli 2024** | 5.03 | 2.84-8.89 | **-** | **-** | **-** |

HR, Hazard Ratio; EPS, Electrophysiological Study; ACA, Aborted Cardiac Arrest; SCD, Sudden Cardiac Death, SCB, Sodium Channel Blockers; ER, Early Repolarization; VA, Ventricular Arrhythmia; RVOT, Right Ventricular Outflow Tract, ΔRP, Difference in Refractory Period

## **Supplementary figure 1:** Forest plot of subgroup analysis depending on the inclusion of patients with history of Aborted Cardiac Arrest in the included studies.


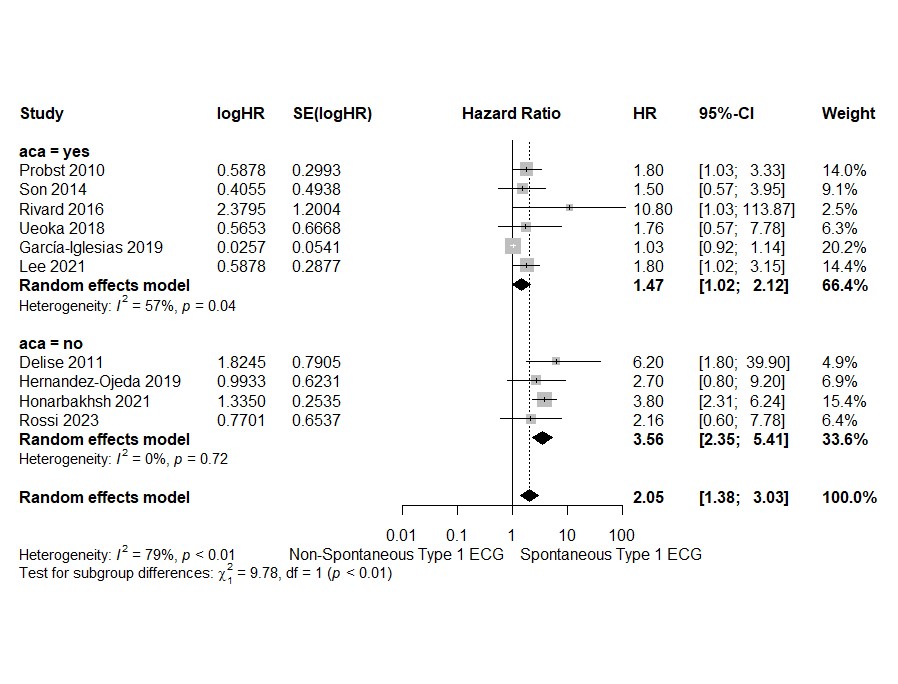


## **Supplementary figure 2:** Forest plot of subgroup analysis depending on the ethnicity of the BrS population in the included studies.


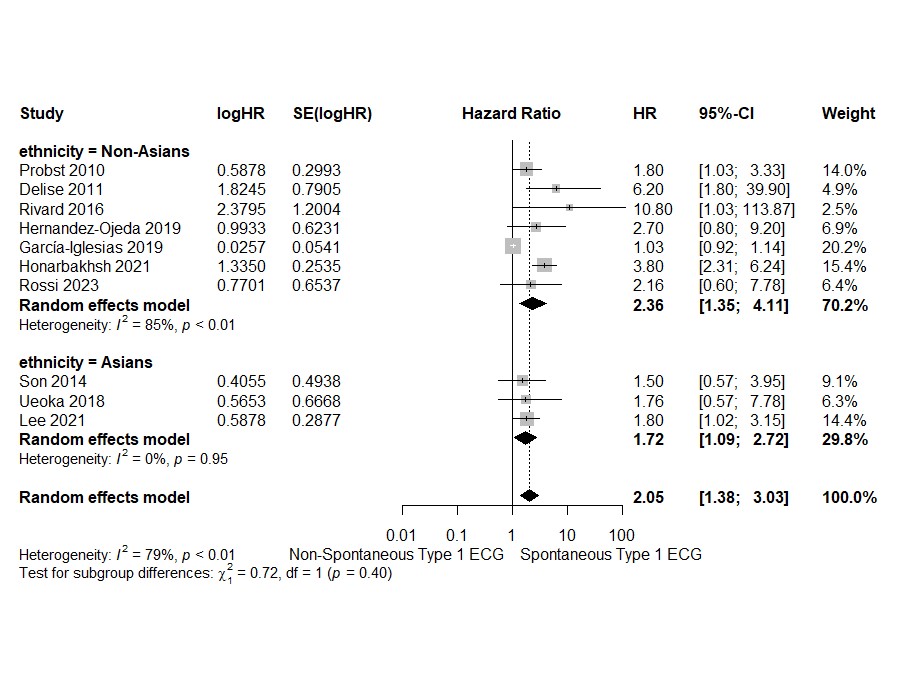


## **Supplementary figure 3:** Forest plot of subgroup analysis depending on the inclusion of syncope as a confounding factor in the included studies.


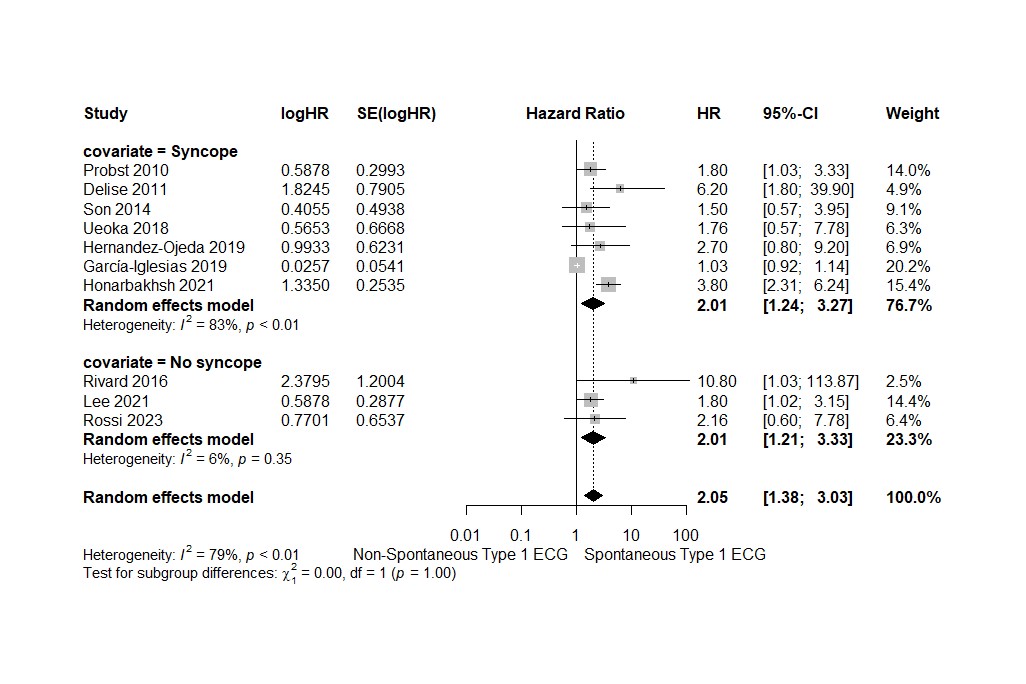


## **Supplementary figure 4:** Leave-one-out sensitivity analysis.


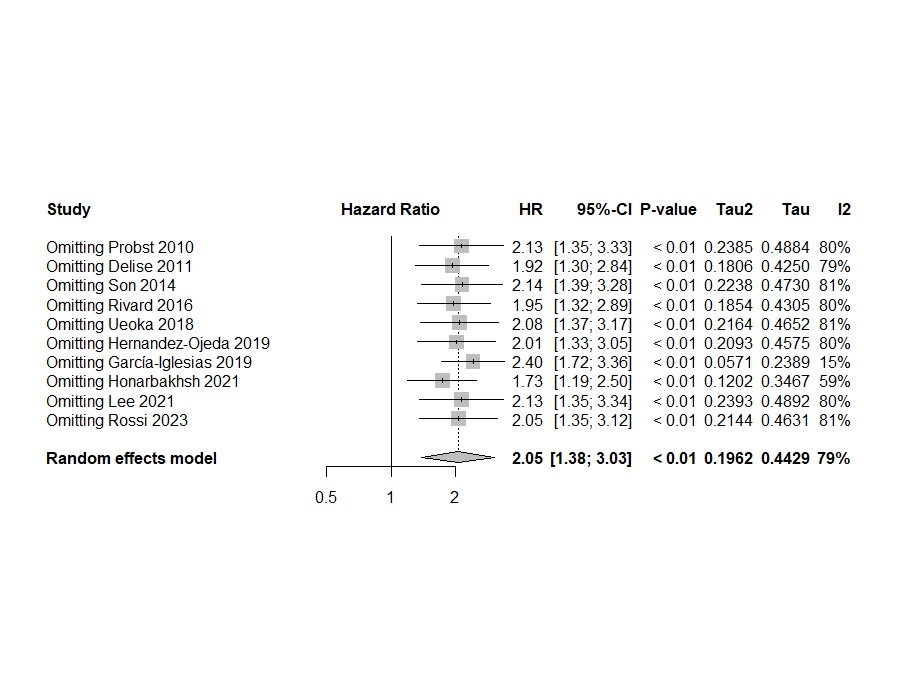


## **Supplementary table 5:** Meta-regression analysis*

| **Factor** | **Number of studies** | **Beta coefficient^1^** | **95% CI** | **P-value^2^** |
| --- | --- | --- | --- | --- |
| Year of publication, per 1 year | 10 | -0.006 | -0.1; 0.09 | 0.91 |
| %Gender male, per 1% | 10 | -0.004 | -0.04; 0.03 | 0.8 |
| Ethnicity, Asians/non-Asians | 10 | 0.29 | -0.61; 1.2 | 0.52 |
| Age, per 1 year | 10 | 0.02 | -0.04; 0.09 | 0.4 |
| %Symptomatic, per 1% | 10 | 0.0003 | -0.01; 0.02 | 0.97 |
| %Spontaneous Type 1 ECG, per 1% | 10 | 0.008 | -0.01; 0.02 | 0.36 |
| %History of Syncope, per 1% | 10 | 0.006 | -0.01; 0.03 | 0.54 |
| %History of aborted cardiac arrest/VA, per 1% | 10 | -0.009 | -0.02; 0.01 | 0.28 |
| Study design, prospective/retrospective | 10 | -0.433 | -1.45; 0.58 | 0.4 |
| 24-h Holter monitoring for detection of type 1 patterns, Yes/No | 10 | 0.05 | -1.57; 1.67 | 0.95 |
| Adjusted for Age, Yes/No | 10 | -0.166 | -1.37; 1.04 | 0.78 |
| Adjusted for Syncope, Yes/No | 10 | -0.548 | -1.26; 0.16 | 0.13 |
| Adjusted for ACA, Yes/No | 10 | -0.292 | -1.2; 0.61 | 0.52 |
| Number of covariates, per 1 | 10 | -0.21 | -0.44; 0.02 | 0.07 |

*Meta-regression was conducted to assess the linear relationship between the explanatory variables and the outcome variables (effect estimates) using a random-effects method.

^1^The regression coefficient (β) and 95% CI describe how the outcome variable (the effect estimate) changes with a unit increase in the explanatory variable (potential moderation effect).

^2^The statistical significance (p value) of the regression coefficient is a test of whether there is a linear relationship between the explanatory variable and the outcome variable. Bold values indicate P<0.05.

## **Supplementary figure 5:** Funnel plot of the studies included in the meta-analysis of aHRs.


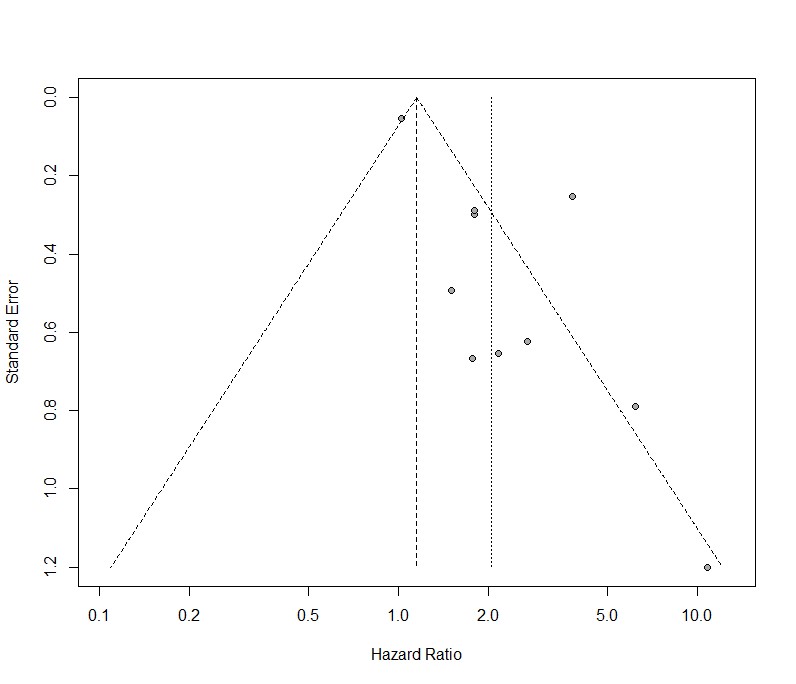


## **Supplementary figure 6.** Funnel plot of trim-and-fill method to correct publication bias.


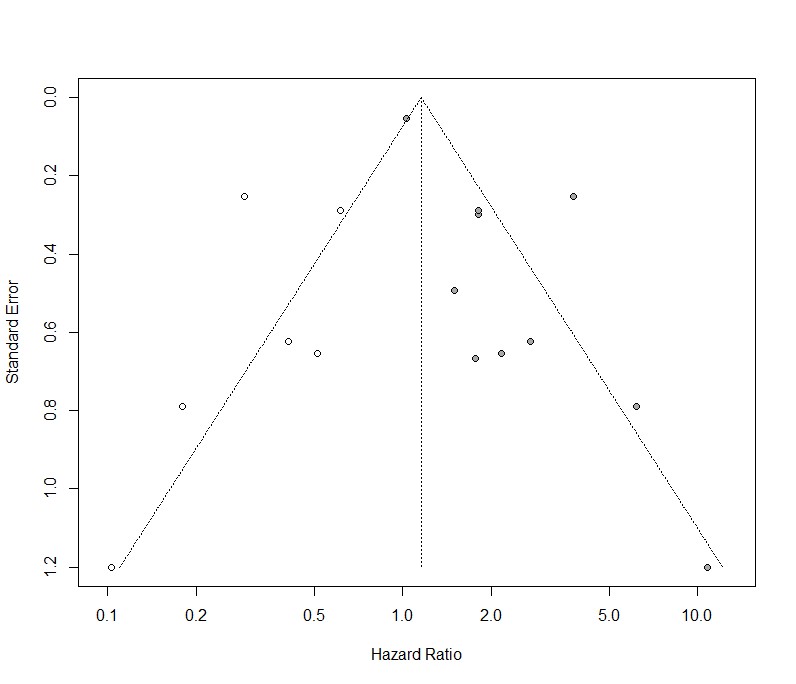


## **Supplementary table 6:** Quality assessment using the Newcastle-Ottawa Scale (NOS)

| **Study** | **Item** **&** **score** | | | | | | | |  |
| --- | --- | --- | --- | --- | --- | --- | --- | --- | --- |
|  | **Representiveness**  **of** **the** **exposed** **cohort（1）** | **Selection** **of** **the**  **non-exposed** **cohort（1）** | **Ascertainment** **of** **exposure**  **（1）** | **Demonstration** **that** **outcome** **of** **interest** **was** **not** **present** **at** **start** **of**  **study（1）** | **Comparability** **of** **cohorts** **on** **the** **basis** **of** **the** **design** **or** **analysis**  **（2）** | **Assessment** **of** **outcome**  **（1）** | **Was** **follow** **up** **long** **enough** **for** **outcomes** **to**  **occur（1）** | **Adequacy** **of**  **follow** **up** **of** **cohorts（1）** | **Total score** |
| **Probst 2010** | + | + | + |  | ++ | + | + | + | 8 |
| **Delise 2011** | + | + | + |  | + | + | + | + | 7 |
| **Priori 2012** | + | + | + |  |  | + | + | + | 6 |
| **Takagi 2013** | + | + | + |  |  | + | + | + | 8 |
| **Son 2014** |  | + | + |  | ++ | + | + |  | 6 |
| **Rivard 2016** | + | + | + |  |  | + | + | + | 6 |
| **Kitamura 2017** | + | + | + |  |  | + | + | + | 6 |
| **Ueoka 2018** | + | + | + |  | + | + | + | + | 7 |
| **Hernandez-Ojeda 2019** |  | + | + |  | + | + | + | + | 7 |
| **García-Iglesias 2019** | + | + | + |  | ++ | + | + | + | 8 |
| **Letsas 2019** | + | + | + |  |  | + | + | + | 8 |
| **Honarbakhsh 2021** | + | + | + |  | ++ | + | + | + | 8 |
| **Lee2021** | + | + | + |  | ++ | + | + | + | 8 |
| **Ishikawa 2021** | + | + | + |  |  | + | + | + | 6 |
| **Rossi 2023** | + | + | + |  |  | + | + | + | 6 |
| **Pannone 2023** | + | + | + |  |  | + | + | + | 6 |
| **Gaita 2023** | + | + | + |  |  | + | + | + | 6 |
| **Santinelli 2024** |  | + | + | + |  | + | + | + | 6 |

## **Supplementary table 7:** Grading of evidence for the primary outcome

| Outcomes | No of participants  (Study) | Relative effect (95% CI) | Certainty of evidence  (GRADE) |
| --- | --- | --- | --- |
| Major arrhythmic events during follow-up in patients with Spontanteous versus non-Spontaneous Type 1 ECG (multivariate model) | 4227  (10 studies) | aHR = 2.05 (1.38; 3.03) | ⊕○○○  **VERY LOW^1^** |
| Major arrhythmic events during follow-up in patients with Spontanteous versus non-Spontaneous Type 1 ECG (univariate model) | 7103  (17 studies) | uHR = 2.97 (2.04; 4.34) | ⊕○○○  **VERY LOW^2^** |
| CI, Confidence Interval; aHR, adjusted Hazard Ratio; uHR, unadjusted Hazard Ratio | | | |
| GRADE Working Group grades of evidence.  High = This research provides a very good indication of the likely effect. The likelihood that the effect will be substantially different is low.  Moderate = This research provides a good indication of the likely effect. The likelihood that the effect will be substantially different is moderate.  Low = This research provides some indication of the likely effect. However, the likelihood that it will be substantially different is high.  Very low = This research does not provide a reliable indication of the likely effect. The likelihood that the effect will be substantially different is very high. | | | |
| ^1^ Substantial statistical heterogeneity across the studies (I^2^=79%), high risk of publication bias  ^2^ Substantial statistical heterogeneity across the studies (I^2^=88%) | | | |
